# MRTF‐A regulates myoblast commitment to differentiation by targeting PAX7 during muscle regeneration

**DOI:** 10.1111/jcmm.16820

**Published:** 2021-08-04

**Authors:** Ruhui Song, Shengnan Zhao, Yue Xu, Jian Hu, Shuangao Ke, Fan Li, Gaohui Tian, Xiao Zheng, Jiajun Li, Lixing Gu, Yao Xu

**Affiliations:** ^1^ Institute of Biology and Medicine College of Life Science and Health Wuhan University of Science and Technology Wuhan Hubei 430081 China; ^2^ Animal Disease Control and Prevention Centre of Chongqing City Chongqing 401120 China

**Keywords:** differentiation, MRTF‐A, muscle regeneration, PAX7, transcriptional regulation

## Abstract

Myocardin‐related transcription factor‐A/serum response factor (MRTF‐A/SRF), a well‐known transcriptional programme, has been proposed to play crucial roles in skeletal muscle development and function. However, whether MRTF‐A participates in muscle regeneration and the molecular mechanisms are not completely understood. Here, we show that MRTF‐A levels are highly correlated with myogenic genes using a RNA‐seq assay, which reveal that MRTF‐A knockdown in C2C12 cells significantly reduces PAX7 expression. Subsequent *in vitro* and *in vivo* data show that MRTF‐A and PAX7 present identical expression patterns during myoblast differentiation and CTX‐induced muscle injury and repair. Remarkably, MRTF‐A overexpression promotes myoblast proliferation, while inhibiting cell differentiation and the expression of MyoD and MyoG. MRTF‐A loss of function produces the opposite effect. Moreover, mice with lentivirus (MRTF‐A) injection possesses more PAX7^+^ satellite cells, but less differentiating MyoD^+^ and MyoG^+^ cells, leading subsequently to diminished muscle regeneration. Our mechanistic results reveal that MRTF‐A contributes to PAX7‐mediated myoblast self‐renewal, proliferation, and differentiation by binding to its distal CArG box. Overall, we propose that MRTF‐A functions as a novel PAX7 regulator upon myoblast commitment to differentiation, which could provide pathways for dictating muscle stem cell fate and open new avenues to explore stem cell‐based therapy for muscle degenerative diseases.

## 1 INTRODUCTION

Dysregulation of muscle homeostasis involved factors can lead to a wide spectrum of pathologic disorders, including sarcopenia, ossification, and muscular dystrophy, which are typically characterized by sustained degeneration and abrogation of muscle tissue.^1^, ^2^ Myogenic progenitor cells, designated as satellite cells (SCs) localized between the basal lamina and the myofibre sarcolemma, play a critical role in the postnatal muscle development and regeneration.^3^, ^4^, ^5^ Emerging evidence revealed that the SC‐mediated muscle repair and the targeted therapeutic strategies not only contribute to the correction of disease aetiology and progression,^6^ but are also necessary for enhancing the regenerative capacity of skeletal muscle in response to traumatic injuries.^7^ SCs normally exist in a quiescent state juxtaposed to the myofibre. The activation of SCs by an exogenous stimulant may lead to the self‐renewal and proliferation, followed by myogenic differentiation events.^8^ However, the imbalance of SCs niche replenishment versus myoblast differentiation is a common phenomenon in pathologic conditions. Identification of key factors that regulate the myogenic lineage could provide a reasonable outlook on the innovative cell‐based solutions for treating muscle damage.

The metabolic properties that maintaining SCs homeostasis such as self‐renewal, proliferation, and differentiation act as positional cues for skeletal muscle regeneration.^9^ The process is tightly regulated by paired box 7 (PAX7) and intrinsic muscle regulatory factors (MRFs).^10^ PAX7, a well‐defined transcriptional regulator, is required for the establishment and function of SCs lineage. Genetic ablation of PAX7 results in a severe deficit loss of SCs pool and blocks the regenerative capacity of myogenesis.^11^, ^12^ Previous *in vivo* studies revealed that PAX7 null mice showed SCs exhaustion due to the restricted self‐renewal competence, which subsequently impeded myoblast differentiation and muscle regeneration, leading to impaired formation of hypaxial somite and limb muscles.^13^, ^14^ Transcriptome and genome sequencing assays revealed that PAX7 can determine the cell fate of myogenic programme by inducing the expression of myogenic factor 5 (Myf5) and myogenic differentiation‐1 (MyoD).^15^, ^16^ Interestingly, declining PAX7 expression in differentiated myoblasts and PAX7 retention in self‐renewing SCs are observed in the transition of SCs into fused myofibre, suggesting the dual effects of PAX7 in muscle progenitors.^17^, ^18^ Although these observations indicate that the PAX7 is implicated in skeletal muscle repair, the factors that regulate PAX7 signalling and the associated mechanisms have yet been identified during muscle regeneration.

The family of Myocardin‐related transcription factors (MRTFs) function as co‐activators of serum response factor (SRF) that contribute to the transcriptional regulation of genes involved in tumour formation and metastasis, cardiac and smooth muscle development, and skeletal muscle metabolism and regeneration via binding to a conserved CArG box sequence (CC(A/T)_6_GG) in the promoter region.^19^, ^20^, ^21^, ^22^ MRTF‐A, a member of the MRTFs family, has previously been linked with physiological process of muscle injury and muscular dystrophy. Knockdown of MRTF‐A in C2C12 myoblasts can block the expression of SRF targeted differentiation involving genes.^23^ The mutant mice harbouring global MRTF‐A deletion are viable, but the skeletal muscles present irreversible hypoplasia.^24^ Previous molecular signalling assays revealed that MRTF‐A and PAX7 showed markedly increased expressions in SCs during muscle regeneration.^25^ Satellite cell‐specific ablation of MEF2 activating motif and SAP domain containing transcriptional regulator (MASTR), another MRTFs member, can impair the myogenic programme by regulating the transcriptional activity of MyoD, which is substantially augmented in the MASTR and MRTF‐A double knockout mice due to aberrant differentiation.^26^ These observations highlight the crucial role of MRTF‐A in skeletal muscle maintenance and regeneration.

Considering the up‐regulated MRTF‐A expression in SCs (PAX7 positive) and its essential role in myofibrillogenesis, the molecular mechanisms by which MRTF‐A regulates these processes are unknown. Thus, in this study, we used the C2C12 myoblast as a cell model to examine the mechanical function of MRTF‐A in the myoblast commitment and cell fate specification during muscle regeneration. This could provide promising evidence to develop new therapeutic target of various skeletal myopathies.

## 2 MATERIAL AND METHODS

### 2.1 Animals

All C57BL/6 mice aged 8‐week‐old used in this study were purchased from Shulaibao Biotech Co. (Wuhan, China). The mice were maintained at 22°C with 30% relative humidity on a 12‐h light/dark cycle and provided food and water *ad libitum*. Animal studies were carried out in accordance with the recommendations in the Guide for the Care and Use of Laboratory Animals from the National Institutes of Health. The protocols were approved by the Institutional Animal Care and Use Committee of Wuhan University of Science and Technology. The cardiotoxin (CTX) (cat. no. 11061‐96‐4, Sigma‐Aldrich, MERCK) and lentivirus injection were performed under anaesthesia, and all efforts were made to minimize animal suffering.

### 2.2 Cell culture and differentiation

The C3H murine skeletal muscle cell line C2C12 myoblasts (American Type Culture Collection, CRL‐1772) were cultured in growth medium (GM), which was consisting of Dulbecco's modified Eagle's medium (DMEM) (cat. no. 10569‐010, Gibco), 10% FBS (cat. no. 10099–141, Gibco) and 1% penicillin‐streptomycin (cat. no. SV30010, Hyclone) at 37°C under a humidified atmosphere with 5% CO_2_. Primary myoblasts were isolated from the limbs of 2‐ to 5‐day‐old C57BL/6 mice. The limb tissue was firstly digested with 1% type II collagenase (cat. no. 17101‐015, Invitrogen), 2.4 U/ml dispase II (cat. no. 42613‐33‐2, Sigma‐Aldrich) and 2.5 mM CaCl_2_ in PBS for 30 min at 37°C. The digested tissue block was screened by nylon mesh filter (100 μm). After centrifugation, the isolated myoblasts were cultured in DMEM supplemented with 30% FBS and 1% penicillin‐streptomycin at 37°C with 5% CO_2_ atmosphere. For differentiation of muscle cells into myotube, the GM was replaced with differentiation medium (DM) containing DMEM supplemented with 2% horse serum (cat. no. 26050088, Gibco) and 1% penicillin‐streptomycin when C2C12 cells and primary myoblasts at 90% confluence. The DM was changed every 24 h and detected in the indicated number of differentiation days. All experimental groups of cell culture were performed at least in triplicate.

### 2.3 Quantitative real‐time PCR

Total RNAs were extracted from C2C12 cells, murine primary myoblasts and skeletal muscle tissue using TRIzol reagent (cat. no. 15596026, Invitrogen). The RNA quantity and quality were detected using Nanodrop™ One (Thermo Scientific). Reverse transcription assay was performed in a 20 μl reaction volume with 2 μg total RNA using the PrimeScript RT Reagent Kit with gDNA Eraser (Perfect Real Time) (cat. no. RR047A, Takara Bio). The SYBR Green kit (cat. no. 4367659, Invitrogen) was used for quantitative real‐time PCR, and the reactions were conducted by a CFX 96™ RealTime Detection System (Bio‐Rad Laboratories) according to the manufacturer's protocol. Glyceraldehyde 3‐phosphate dehydrogenase (*GAPDH*) was used as a candidate housekeeping gene for normalization. The relative expression of all measured genes was normalized to the *GAPDH* level, and the fold change of gene expression was calculated according to the 2^–ΔΔCq^ method. All qPCR assays were repeated in triplicate. Primer sequences for qPCR are presented in Table S1.

### 2.4 Western blot analysis

Western blot was performed to detect the protein level of candidate genes. In detail, the control or treated C2C12 cells were rinsed with PBS three times and then lysed with 200 μL radioimmunoprecipitation assay (RIPA) buffer (20 mM Tris‐HCl, 1% SDS, 150 mM NaCl, 1% sodium deoxycholate and 1% Triton X‐100) containing 1 mM PMSF and 0.02% protease phosphatase inhibitors. For tissues, a total of 30 mg tibialis anterior muscle from C57BL/6 mice was homogenized with 600 μl RIPA buffer. Cells or tissue extracts were centrifuged at 14,000 ***g*** and 4°C for 15 min to remove the insoluble matter. The protein concentration in the supernatants was quantified by BCA detection assay. Aliquots containing 20 μg total protein were diluted in 5× loading buffer and separated in 10% SDS‐PAGE. The dispersive proteins in gel were then transferred to PVDF membrane, which was blocked in Tris saline with Tween (TBST) containing 5% skim milk and Tween 20 for 2 h at room temperature. Next, the membrane was incubated with primary antibodies at 4°C overnight in blocking buffer, followed by incubation with horseradish peroxidase (HRP)–conjugated secondary antibodies for 2 h at room temperature. Finally, the protein bands were detected using chemiluminescent HRP kit (cat. no. WBKLS0500, Merck KGaA, Darmstadt, Germany). The information of all primary antibodies was as follows: rabbit anti‐MRTF‐A (1:1000, cat. no. 14760S, Cell Signaling Technology), rabbit anti‐PAX7 (1:1500, cat. no. abs124153, Absin), mouse anti‐MyoD (1:500, cat. no. NBP2‐32882, Novus), mouse anti‐MyoG (1:1000, cat. no. M5815, Sigma‐Aldrich), rabbit anti‐β‐actin (1:1000, cat. no. 3700S, Cell Signaling Technology) and mouse anti‐GAPDH (1:1000, cat. no. sc‐47724, Santa Cruz). Secondary antibodies were HRP‐conjugated anti‐rabbit or anti‐mouse IgG antibodies.

### 2.5 RNA‐Seq and data analysis

Total RNA of the C2C12 cells with stable transfected sh‐MRTF‐A or sh‐control was isolated using TRIzol reagent. For RNA sequencing, the RNA quality and integrity were examined using Bioanalyzer 2100 and RNA 1000 Nano LabChip Kit (Agilent), and the sample with RIN (RNA Intergrity Number) less than 7 was excluded from the subsequent assay. The mRNA was enriched from the qualified RNA using oligo (dT) beads and then fragmented into short sequences using fragmentation buffer. After cDNA library preparation, RNA‐seq was performed by Illumina HiSeq 4000 (paired‐end, 150 bp, PE150). Raw data files (.fastq) were mapped to the mouse reference genome Mus_musculus GRCm38 using HISAT package (http://ccb.jhu.edu/software/hisat2). The aligned reads were assembled using StringTie software. Then, all transcriptomes were merged to reconstruct a comprehensive transcriptome using perl scripts. StringTie and EdgeR were used to estimate the differentially regulated genes of all transcripts by calculating FPKM. The differentially expressed genes were determined with log2 (fold change) >1 or log2 (fold change) <−1 and with statistical significance (*p* value < 0.05) by R package.

### 2.6 Dual‐Luciferase reporter assay

Two CArG box motifs were predicted in promoter region of the *PAX7* gene. According to this regulatory region, a ~619‐bp sequence containing point‐mutated CArG‐1/2 or cut‐off CArG‐1/2 was amplified and inserted into the luciferase reporter vector pGL3‐Basic (cat. no. E1751, Promega, Madison, WI) using *Kpn* I and *Hin*d III double digestion assay. For promoter activity detection, C2C12 cells were seeded in a 48‐well plate, and 400 ng total plasmids containing pGL3‐CArG and pRL‐TK (cat. no. E2231, Promega, Madison, WI) were transfected using Lipfectamine™ 3000 reagent (cat. no. L3000015, Invitrogen, Carlsbad, CA) when the C2C12 cells reached a 70% confluence. Further culture for 48 h, the C2C12 cells were lysed, and the luciferase activity was determined using Dual‐Luciferase Reporter Assay System (cat. no. E1910, Promega, Madison, WI) according to the manufacturer's protocol. The promoter activity for each sample was detected by normalizing the fluorescence intensity of firefly luciferase to renilla luciferase and then compared with the control group. In addition, the co‐transfections of pGL3‐CArG, pRL‐TK, plus either pCDH‐MRTF‐A or pLKO.1‐sh‐MRTF‐A into C2C12 cells were also established to investigate the effect of MRTF‐A on promoter activity of the *PAX7* gene.

### 2.7 Cell proliferation assay

The proliferation of the stable C2C12 cell with overexpressed MRTF‐A or interfered MRTF‐A was detected by EdU Cell Proliferation Assay Kit (cat. no. C10310, RiboBio, Guangzhou, China) and Cell Counting Kit‐8 (CCK‐8) reagent (cat. no. C0042, Beyotime, Shanghai, China). First, the treated and control C2C12 cells were separately seeded in a 24‐well plate and cultured in GM for approximately 70% confluence, and then, the GM was replaced with fresh medium containing 50 μM EdU for further 2.5‐h incubation. The EdU immunostaining was performed following the manufacturer's instruction, and the images were recorded using Olympus FV3000 confocal microscope (Olympus, Tokyo, Japan). The ratio of EdU‐positive C2C12 cells was calculated by normalizing the total nuclei. For CCK‐8 assay, the treated and control C2C12 cells were separately seeded in a 96‐well plate and cultured in GM for 24, 48 and 72 h. A volume of 10 µL CCk‐8 reagent was added in each plate for 24‐h intervals. After 1‐h incubation, optical density was measured at 450 nm on a SpectraMax i3 Multimode Reader (Molecular Devices).

### 2.8 Flow cytometry

Apoptosis of C2C12 cells under different treatment was measured using FITC Annexin V Apoptosis Detection Kit (cat. no. 556547, BD Biosciences) through flow cytometry assay. Cells were seeded at the density of 1 × 10^6^ cells/well into 6‐well plate and cultured for 24 h. Then, the cells were washed with PBS for three times, digested with trypsin and suspended in 1 × binding buffer. The suspended cells (1 × 10^5^ cells) were incubated with 5 µl of FITC Annexin V and 5 µl of PI for 15 min at room temperature in the dark. Afterwards, the dispersive cells were detected by flow cytometry, and each group had three independent experimental replicates. Four stages of C2C12 myoblasts were detected: live (Annexin V^−^/PI^−^), early apoptosis stage (annexin V^+^/PI^−^), late apoptosis stage (Annexin V^+^/PI^+^) and necrocytosis (Annexin V^−^/PI^+^).

### 2.9 Plasmid construction and lentivirus infection

According to the sequence of MRTF‐A (GenBank ID: 223701), the coding sequence (CDS) region was amplified and cloned into the *Xba* I and *Eco*R I sites of pCDH‐vector‐v5 (Promega) to construct pCDH‐MRTF‐A. For MRTF‐A knockdown, the shRNA sequences (Target: CATGGAGCTGGTGGAG AAGAA) were annealed and inserted into pLKO.1 vector (Sigma‐Aldrich, MERCK) using the *Age* I and *Eco*R I restriction sites (named as shRNA‐MRTF‐A). The primers used for vector construction were shown in Table S1. The constructed plasmids pCDH‐MRTF‐A or shRNA‐MRTF‐A, and packaging plasmids (pCMV‐VSV‐G and pCMV‐Gag‐Pol) were co‐transfected in HEK‐293T cells by Lipofectamine 3000 reagent (cat. no. L3000015, Invitrogen). After 72‐h incubation, the medium was collected, and the virus titre was determined using Lenti‐Pac™ HIV qRT‐PCR Titration Kit (cat. no. HPR‐LTK‐050, GeneCopoeia) according to the manufacturer's protocol. C2C12 cells were infected with the lentivirus (MOI = 5) and 8 μg/ml polybrene. Then, the infected C2C12 cells were screened using 2 μg/ml puromycin for 2 weeks. The remaining C2C12 cells were evaluated for overexpression or knockdown efficiency of the MRTF‐A by qPCR and Western blot assay.

### 2.10 Muscle injury and regeneration

Muscle injury in mice was induced with CTX (cat. no. 11061‐96‐4, Sigma‐Aldrich, MERCK) injection. In detail, the ketamine (10 mg/kg) plus xylazine (1 mg/kg) was firstly injected into enterocoelia of C57BL/6 mice aged 8‐week‐old for anaesthesia. Then, muscle injury was induced by injection of 50 μl of 10 µM CTX (diluted in PBS) into the mid‐belly of the right tibialis anterior (TA) muscle. Meanwhile, an equal amount of PBS was injected into the left TA muscle of each mouse as an internal control. At respective 1, 2, 3, 5, 7, 10 and 14 days after treatment, mice were euthanized and both TA muscles were harvested to assess the degree of muscle regeneration and repair. In addition, the TA muscles from 5 C57BL/6 mice aged 8‐week‐old were injected with lentivirus of control and MRTF‐A overexpression, respectively. After one week of lentivirus injection, muscle injuries of these mice were induced by injection of 50 μl of 10 µM CTX. After 5 days of CTX injection, the mice were euthanized and all TA muscles were collected for further detection.

### 2.11 Satellite cell isolation

The TA muscles of C57BL/6 mice were dissected and digested with collagenase/dispase, and the satellite cells were enriched by magnetic‐activated cell sorting. The isolated satellite cells were cultured with growth medium (DMEM with 20% FBS supplemented with 10 ng/mL basic fibroblast growth factor) or differentiation medium (DMEM with 2% horse serum).

### 2.12 Haematoxylin‐eosin staining

The injured or regenerated murine TA muscles at different stages were firstly immersed in 4% paraformaldehyde for 30 min at room temperature, and then, the tissues were dehydrated in paraffin. The murine muscle sections were prepared from paraffin‐embedded tissues and stained with HE Staining reagents (cat. no. C0105S, Beyotime, Shanghai, China) following the manufacturer's instructions. The HE staining images of TA muscle sections were recorded using Olympus BX53 microscope (Olympus, Tokyo, Japan).

### 2.13 Immunohistochemistry assay

Immunohistochemistry assay was performed for the paraffin sections of murine TA muscles by UltraSensitive™ SP (Mouse/Rabbit) IHC Kit (cat. no. KIT‐9710, Maxim, Fuzhou, China). In detail, the muscle sections were deparaffinized and rehydrated using xylene and ethyl alcohol with different concentrations, and antigen retrieval was performed by boiling the samples in the retrieval solution (0.1 M citric acid/sodium citrate) for 15 min. Then, the samples were blocked for 1 h using normal non‐immunone serum at 37°C and incubated with primary antibodies directed against MRTF‐A (1:200, cat. no. orb1706, Biorbyt), PAX7 (1:500, cat. no. abs124153, Absin) and Desmin (1:200, cat. no. ab15200, Abcam) overnight at 4°C. Sections were then incubated with biotin‐labelled secondary antibodies for 10 min at room temperature. After washing and haematoxylin restaining, the stained slides were observed by Olympus BX53 microscope (Olympus, Tokyo, Japan).

### 2.14 Immunofluorescence staining

Immunofluorescence assay was performed to determine the effects of different treatment on cell commitment towards differentiation. Briefly, myoblast cells grown on glass coverslips were firstly washed with PBS for three times and then fixed with 4% formaldehyde for 20 min and permeabilized in 0.2% Triton X‐100 for 15 min. Thereafter, the cells were blocked non‐specific binding by incubating with 5% bovine serum albumen (BSA) in PBS for 1 h and incubated with primary antibodies diluted in 1% BSA at 4°C overnight, followed by incubation with the corresponding fluorophore‐conjugated secondary antibodies (1:400, cat. no. BA1101/BA1032, FITC/Cy3‐labelled IgG, Boster, China) in dark for 1 h at room temperature. After washing with PBS for five times, cell nuclei were stained with 4′,6‐diamidino‐2‐phenylindole (DAPI) for 20 min in dark. The primary antibodies used were as follows: PAX7 (1:200, cat. no. sc‐81648, Santa Cruz), MyoD (1:300, cat. no. NB100‐56511, Novus), MyoG (1:500, cat. no. M5815, Sigma‐Aldrich) and MyHC (cat. no. MF20, 1:500, DSHB). The coverslips were imaged using Olympus FV3000 confocal microscope (Olympus, Tokyo, Japan). All immunostaining was performed in three independent experiments, and at least six views were captured from each cell well.

### 2.15 Electrophoretic Mobility Shift Assay (EMSA)

According to the CArG box sequences of *PAX7* promoter, we designed the specific probes for EMSA detection. The double‐strand DNA probes containing the CArG box 1 were Probe1‐F (5′‐AAACCCAAATTTGCCAGTGAAGAGCTACCAA AC‐3′) and Probe1‐R (5′‐GTTTGGTAGCTCTTCACTGGCAAATTTGGGTTT‐3′), as well as Probe2‐F (5′‐CTGCCATACCAGGAGGGTGTTGGTGGGGGTAG‐3′) and Probe2‐R (5′‐CTACCCCCACCAACACCCTCCTGGTATGGCAG‐3′) for CArG box 2. Both biotin end‐labelled probes and unlabelled oligonucleotides were synthesized by Genewiz (Suzhou, China). EMSA was performed using the Chemiluminescent Nucleic Acid Detection Module Kit (cat. no. 89880, Thermo Scientific) according to the manufacturer's instructions. In detail, the nuclear extracts of C2C12 cells were prepared for the next step. Protein‐DNA binding reactions were conducted in 20 μl volume containing nuclear extracts and biotin‐labelled probes, while biotin‐labelled probes without nuclear extracts were used as the negative control, and a combination of nuclear extracts, biotin‐labelled probes and unlabelled probe (200×) were incubated for competition. The mixed reaction products were separated by a 6% polyacrylamide gel, and then, the protein‐DNA complexes were transferred to a positively charged nylon membrane. Finally, the membrane was cross linked and detected by chemiluminescence.

### 2.16 Chromatin immunoprecipitation (ChIP) assay

ChIP assay was performed using SimpleChIP^®^ Enzymatic Chromatin IP Kit (Agarose Beads) (cat. no. 9003S, Cell Signaling Technology) according to the manufacturer's protocols. Firstly, the C2C12 cells were incubated with 1% formaldehyde to crosslink nuclear proteins and chromatin for 10 min at room temperature. After treatment with PBS and PIC, the cell nucleus was extracted by centrifugation at 4°C, and then, the nuclear chromatin was sheared to small fragments (200–300 bp) by 0.5 μL micrococcal nuclease treatment and sonication. Different antibodies including MRTF‐A (1:200, cat. no. 14760S, Cell Signaling Technology), Normal Rabbit IgG (negative control) and Histone H3 Antibody (positive control) were added to immunoprecipitate the sheared DNA overnight at 4°C. The pulled down DNA from the treated samples and the input was analysed by direct PCR and SYBR Green qPCR assay. Primers used for ChIP are given in Table S1.

### 2.17 Statistical analysis

All data are obtained based on at least three independent experiments for each treatment. Student's *t* test was used to analyse the statistical difference between two groups. The significant variation among multiple groups was determined by one‐way analysis of variance with Tukey's post hoc test. Data are expressed as the mean ± standard error of the mean (SEM). Data analysis was performed using SPSS software (version 20.0; Illinois). Statistical significance was achieved when the value of *p* < 0.05 (*p < 0.05, **p < 0.01 and ***p < 0.001).

## 3 RESULTS

### 3.1 MRTF‐A knockdown impacts the skeletal muscle regulation involving genes

**FIGURE 1:**
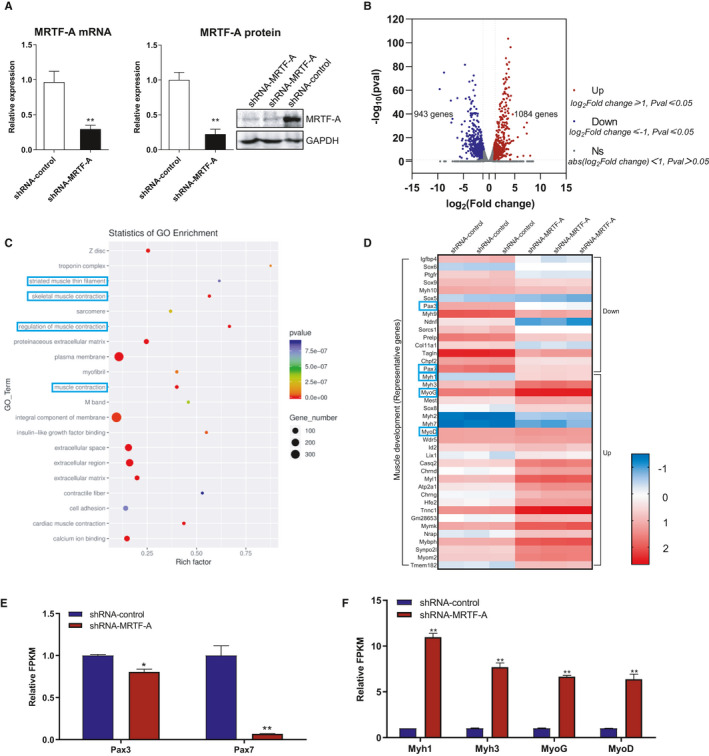
RNA‐seq results reveal MRTF‐A knockdown down‐regulates/up‐regulates the skeletal muscle regulation involving genes in C2C12 cells. (A) The mRNA and protein levels of MRTF‐A in C2C12 cells transfected with shRNA lentivirus targeting MRTF‐A as compared to the shRNA‐control group. (B) Volcano plot of differentially expressed genes between sh‐MRTF‐A and shRNA‐control in C2C12 cells as determined by RNA‐seq. (C) Statistics of GO Enrichment analysis to categorize the pathways that are significantly altered upon MRTF‐A knockdown. The striated muscle thin filament, skeletal muscle contraction, regulation of muscle contraction and muscle contraction signalling are highlighted. (D) The heat map analysis showing the differentially regulated skeletal muscle development genes, data were presented as log_2_(FPKM+1). (E) Validation of identified muscle cell self‐renewal–related genes through FPKM (Reads Per Kilobase of exon model per Million mapped reads, FPKM ≥1) in MRTF‐A knockdown or control muscle cells. (F) Validation of identified muscle cell differentiation related genes through FPKM. *p < 0.05, **p < 0.01

To investigate the differential effects of MRTF‐A on a global transcriptome changes in muscle cells, we first constructed the C2C12 cells with stable MRTF‐A knockdown (shRNA‐MRTF‐A) or control (shRNA‐control), and total RNA was isolated and subjected to RNA‐seq analysis. The mRNA and protein level of the *MRTF*‐*A* gene in shRNA‐MRTF‐A‐transfected C2C12 cells were significantly reduced to 29.4% and 22.3% of the level in shRNA‐control group, respectively, as determined prior to sequencing (Figure 1A). A volcano plot analysis revealed that a total of 1084 up‐regulated and 943 down‐regulated genes were determined in MRTF‐A knockdown C2C12 cells based on the significance criterion (*p*‐value) (Figure 1B). Next, we performed GO enrichment analysis, and the results showed that the biological processes including striated muscle thin filament and skeletal muscle contraction were enriched (Figure 1C). To further identify the functional genes related to skeletal muscle development, we listed 25 representative up‐regulated and 15 down‐regulated genes as a heat map. As shown in Figure 1D, the expression levels of *PAX3* and *PAX7* were decreased, while the other myogenic genes including *Myh1*, *Myh3*, *MyoD and MyoG* were increasingly expressed in response to MRTF‐A knockdown. The significant expression alterations of these genes were demonstrated according to FPKM value in shRNA‐MRTF‐A and shRNA‐control transfected C2C12 cells (Figure 1E and F). Previous studies revealed that PAX7 and PAX3 play crucial roles in muscle stem cell quiescence and activation, and Myh1, MyoD and MyoG contribute to skeletal muscle cell differentiation.^27^, ^28^ Therefore, the RNA‐seq data suggest that MRTF‐A may participate in skeletal muscle development and regeneration by regulating the corresponding target genes.

### 3.2 Consistent expression of MRTF‐A and PAX7 during myoblast differentiation and muscle regeneration

**FIGURE 2:**
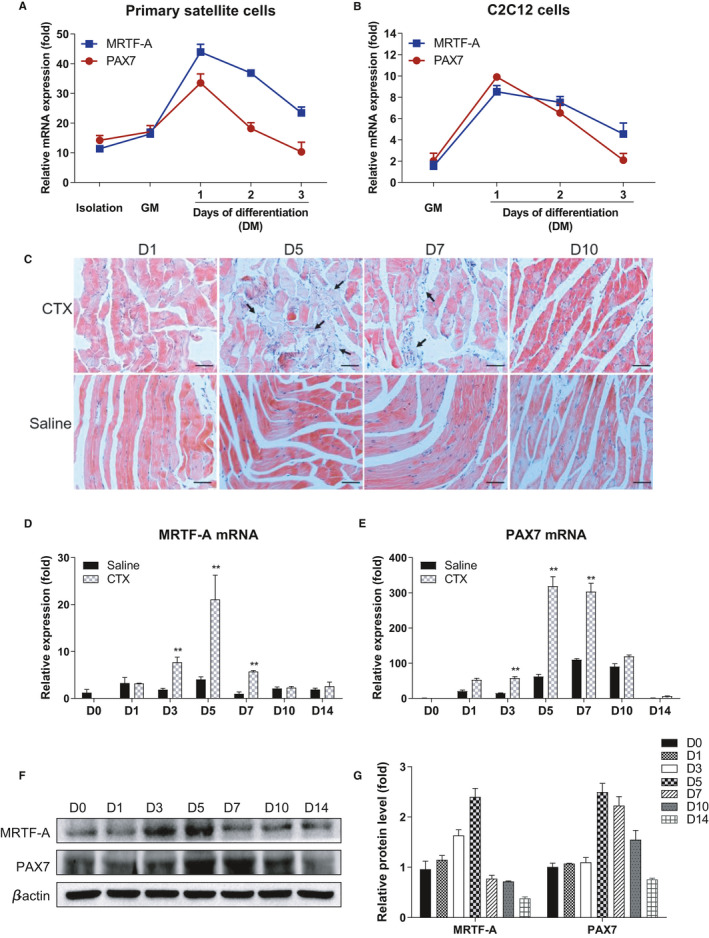
Up‐regulation of MRTF‐A and PAX7 during muscle cell differentiation and muscle regeneration. (A) MRTF‐A and PAX7 presented the same expression patterns during satellite cells (SCs) differentiation. The qPCR assay was conducted using sorted SCs, SCs maintained in growth medium (GM), and SCs at days 1, 2 or 3 of differentiation in differentiation medium (DM). (B) The mRNA expression of the *MRTF*‐*A* and *PAX7* genes in differentiating C2C12 cells. (C) Haematoxylin‐eosin stain was performed to assess the muscle repair after CTX injection for 1, 5, 7 and 10 days. NS represented the normal saline‐treated group. Scale bar, 50 μm. (D) The MRTF‐A mRNA expression in NS control and muscle tissue after CTX injection for 0, 1, 3, 5, 7, 10 and 14 days. (E) The PAX7 mRNA expression in NS control and muscle tissue after CTX injection for 0, 1, 3, 5, 7, 10 and 14 days. (F) The protein level of the MRTF‐A and PAX7 in CTX‐injected tissues. (G) The densitometric quantification analysis of three independent Western blot experiments. The β‐actin was used to serve as a loading control. **p < 0.01

The other family members of the Myocardin transcription factors are predominantly implicated in the regulation of vascular smooth muscle and skeletal muscle differentiation.^29^, ^30^ Considering the inhibitory role of MRTF‐A knockdown in PAX7 expression, we ask whether MRTF‐A associates with myoblast cell commitment and muscle regeneration. First, we isolated the SCs from murine skeletal muscle and examined the expression pattern of *MRTF*‐*A* and *PAX7* during proliferation and differentiation of SCs (Figure 2A) and C2C12 cells (Figure 2B) *in vitro*. As expected, *MRTF*‐*A* and *PAX7* expression was elevated when SCs or C2C12 cells were activated to proliferate, whereas their expressions declined when the cells were induced to differentiate into myotubes. Moreover, to address if MRTF‐A, similar to PAX7, is involved in muscle regeneration *in vivo*, we used the mouse model for muscle degeneration and regeneration by CTX injection. Severe damage was observed at 5 days, and the repair was almost completed at 10 days (Figure 2C). Consistently, we detected the highest mRNA expression levels of *MRTF*‐*A* and *PAX7* at 5 days, which then gradually diminished from 7 days to 14 days post–injection (Figure 2D and E). Similar protein levels were also confirmed by Western blot (Figure 2F and G) and immunohistochemistry (Figure S1). In addition, we isolated the SCs from murine TA muscles at 1, 5 and 10 days post–CTX injection, respectively, and performed immunofluorescence for PAX7, MyoD and MyoG. The results showed increased numbers of PAX7^+^ SCs at 5 days but reduced PAX7^+^ SCs at 10 days. Conversely, declining MyoD^+^ and MyoG^+^ SCs were observed at 5 days, however, they climbed up to a high level at 10 days (Figure S2A). The results were also verified by Western blot (Figure S2B). Based on these findings, we conclude that MRTF‐A is involved in the early muscle repair along with SCs activation and proliferation and displays a consistent expression with the SCs marker PAX7.

### 3.3 Forced expression of MRTF‐A enhances myoblast proliferation

**FIGURE 3:**
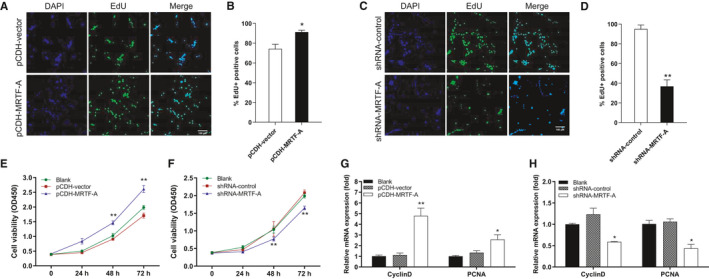
MRTF‐A promotes the proliferation of myoblast. (A) C2C12 myoblasts were transfected with pCDH‐vector or pCDH‐MRTF‐A, and cell proliferation was assessed using 5′‐Ethynyl‐2′‐deoxyuridine (EdU) assay. The scale bar represents 100 μm. (B) The percentage of EdU‐positive cells was analysed in figure (A). (C) The EdU assay was used to detect the cell proliferation of C2C12 cells that was transfected with shRNA‐control or shRNA‐MRTF‐A. The scale bar represents 100 μm. (D) The percentage of EdU‐positive cells was analysed in figure (C). Cell proliferation was detected using the cell counting kit‐8 (CCK‐8) assay in C2C12 cells with MRTF‐A overexpression (E) and MRTF‐A knockdown (F) comparing to control groups. (G, H) The mRNA levels of the proliferation marker gene *CyclinD1* and *PCNA* were quantified using qPCR. Data are presented as means ± SEM for three independent experiments. *p < 0.05, **p < 0.01.

The acceleration of MRTF‐A level in early injury indicates its potential role in muscle cell expansion. To assess whether MRTF‐A contributes to the proliferation of undifferentiated myoblasts, we generated the C2C12 cells with stable MRTF‐A overexpression (pCDH‐MRTF‐A) and MRTF‐A knockdown (shRNA‐MRTF‐A), as well as the control groups pCDH‐vector and a scrambled shRNA‐control, respectively. EdU assay was used to evaluate cell proliferation, and the results showed that the MRTF‐A overexpression significantly promoted mitotic activity in comparison to the control C2C12 cells (Figure 3A and B). The effect was reversed in MRTF‐A knockdown C2C12 myoblasts (Figure 3C and D). Cell proliferation was also analysed using the CCK‐8 assay. Similarly, we found that exogenous overexpression of MRTF‐A resulted in a significant increase of C2C12 cell proliferation (Figure 3E), while down‐regulation of MRTF‐A caused decreased proliferation (Figure 3F). Besides the cell phenotypic characteristics, elevated expression of the marker genes for cell proliferation (*Cyclin D1* and *PCNA*) was investigated in C2C12 cells with MRTF‐A overexpression compared to the knockdown group, which were detectible at the mRNA level (Figure 3G and H). In addition, we found that MRTF‐A overexpression or knockdown had no effects on C2C12 cell apoptosis (Figure S3A and B). Collectively, these data indicate that MRTF‐A promotes the myoblast proliferation.

### 3.4 MRTF‐A regulates myoblast heterogeneity by enhancing PAX7 levels

**FIGURE 4:**
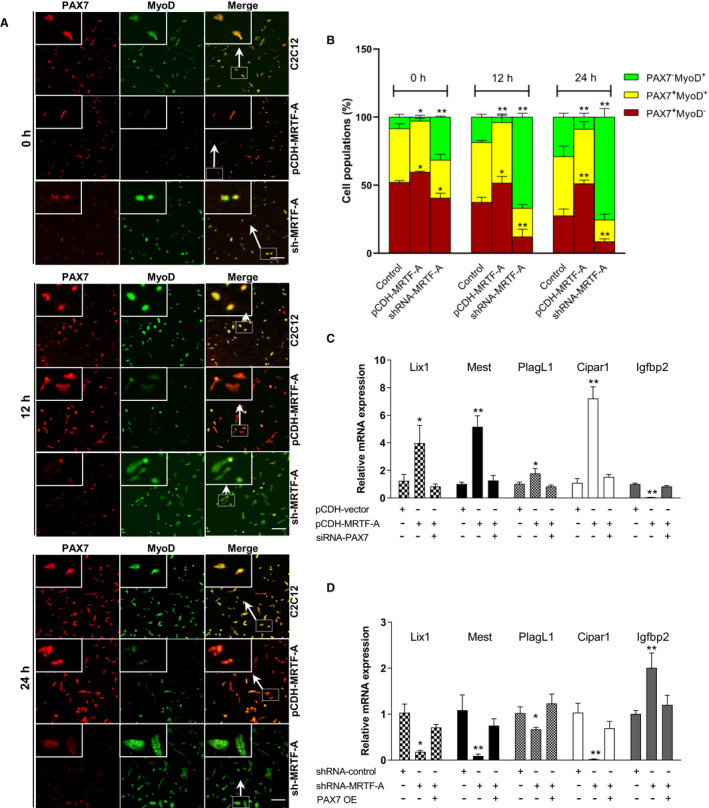
MRTF‐A overexpression inhibits myoblast commitment towards differentiated cells. (A) Coimmunostaining for PAX7 (red) and MyoD (green) of C2C12 control cells, MRTF‐A knockdown and MRTF‐A overexpression C2C12 cells at 0, 12 and 24 h. The representative images of each group are shown. The inset photograph (large box) represented the higher magnification (200×) of the C2C12 cells from the small box. The scale bar represents 100 μm. (B) Percentage of PAX7^+^/MyoD^−^, PAX7^+^/MyoD^+^ and PAX7^−^/MyoD^+^ cells were analysed during onset of the differentiation process in 15 different microscopic fields. The data show mean values of the percentage of cells on three different slides, error bars represent standard error of the mean. The experiment was repeated twice with similar results. (C) The mRNA expression of potential PAX7‐target genes in C2C12 cells. C2C12 cells were divided into three groups: stably transfected pCDH‐vector, stably transfected pCDH‐MRTF‐A, transfected siRNA‐PAX7 in stably transfected pCDH‐MRTF‐A cells. (D) The mRNA expression of potential PAX7‐target genes by rescuing of the MRTF‐A knockdown C2C12 cell with PAX7 overexpression (PAX7 OE). C2C12 cells were divided into three groups: stably transfected shRNA vector, stably transfected shRNA‐MRTF‐A, transfected pCDH‐PAX7 in stably transfected shRNA‐MRTF‐A cells. *p < 0.05, **p < 0.01

The three typical clusters of muscle cell progeny including self‐renewing (PAX7^+^/MyoD^−^), proliferating (PAX7^+^/MyoD^+^) and differentiating (PAX7^−^/MyoD^+^) are commonly used for evaluating cell heterogeneity.^31^ To further explore whether MRTF‐A regulates the myoblast commitment towards differentiation, the coexpression of PAX7 and MyoD was detected during the first 24 h of the differentiation process (Figure 4A). In control C2C12 cells at 0 h, the proportions of PAX7^+^/MyoD^−^, PAX7^+^/MyoD^+^ and PAX7^−^/MyoD^+^ cell populations were 52.3%, 39.6% and 8.1%, respectively. In MRTF‐A overexpressed cells, the pool of progenitors, PAX7^+^/MyoD^−^, was significantly replenished among the three time points. Moreover, the population of PAX7^−^/MyoD^+^ cells in MRTF‐A knockdown group was drastically increased in favour of PAX7^+^/MyoD^−^ and PAX7^+^/MyoD^+^ cells (Figure 4B). We next assessed whether MRTF‐A regulates transcription profile of PAX7 and its downstream genes. The results showed that PAX7 and its target genes including *Lix1*, *Mest*, *PlagL1* and *Cipar1* were significantly increased in MRTF‐A overexpressed cell, while *Igfbp2*, a gene that is negatively regulated by PAX7, was reduced. The levels of these genes can be returned by simultaneous knockdown of PAX7 (Figure 4C). Moreover, the effects were reversed when PAX7 was overexpressed in MRTF‐A knockdown cell (Figure 4D). Together, our results demonstrate that the MRTF‐A is required for PAX7‐mediated myoblast commitment.

### 3.5 Forced expression of MRTF‐A inhibits myoblast cell differentiation

**FIGURE 5:**
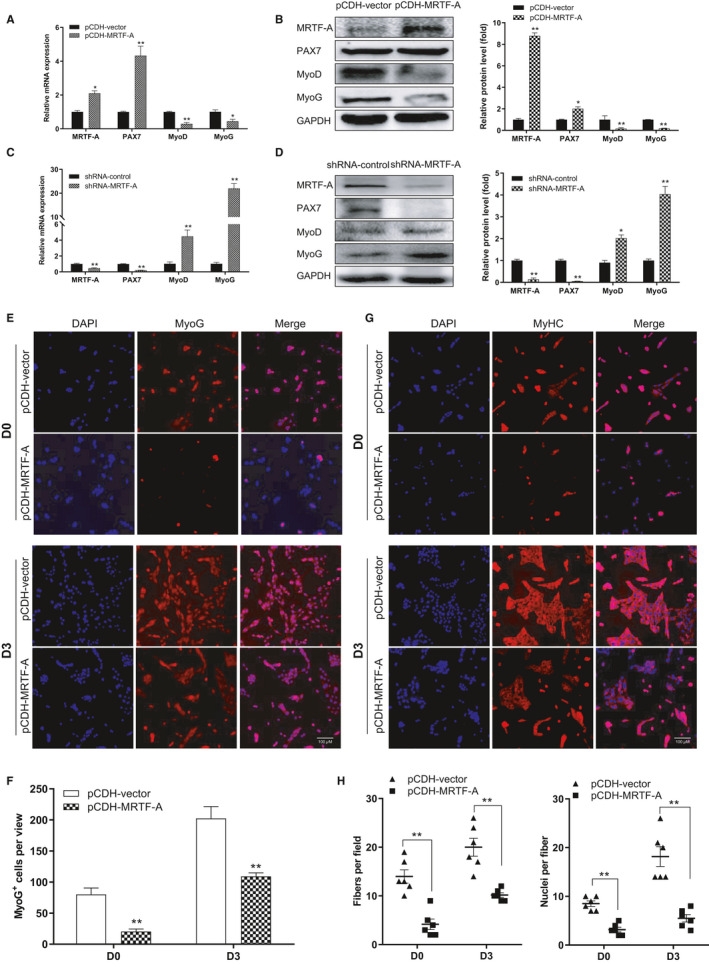
MRTF‐A inhibits the differentiation of myoblast. (A) The mRNA expression of MRTF‐A, PAX7, MyoD and MyoG in C2C12 cells transfected with pCDH‐vector or pCDH‐MRTF‐A. (B) Western blot for MRTF‐A, PAX7, MyoD and MyoG in C2C12 cells transfected with pCDH‐vector or pCDH‐MRTF‐A. (C, D) The mRNA and protein level of MRTF‐A, PAX7, MyoD and MyoG in C2C12 cells transfected with shRNA‐control and shRNA‐MRTF‐A. The densitometric quantification was analysed from three independent Western blot experiments (B, D). GAPDH expression was analysed to ensure equal loading of samples. (E) The differentiation of C2C12 cells stably overexpressing MRTF‐A was examined by staining for MyoG after 0 d (D0) and 3 d (D3) of culture in DM. Cells with an empty vector as the control. (F) The graph shows MyoG‐positive cells as a proportion of total cell number (shown by DAPI staining) for MRTF‐A‐overexpressing cells compared to control cells. (G) MyHC immunocytochemistry (red) for MRTF‐A overexpressing cells and control cells (pCDH‐vector) at D0 and D3 of differentiation. (H) Number of fibres per field of view and number of nuclei per fibre in images used for counting in (G), shown relative to control cells, *p < 0.05, **p < 0.01

Increased PAX7^−^/MyoD^+^ cell population is required to initiate myogenic differentiation. Since the number of PAX7^−^/MyoD^+^ cells was negatively correlated with MRTF‐A expression, we next examined whether MRTF‐A can regulate the differentiation of myoblasts into myotubes. First, we confirmed the overexpression of MRTF‐A in C2C12 cells at the mRNA and protein level (Figure 5A and B). In addition, the increased PAX7 and decreased expressions of MyoD and MyoG were also identified in response to MRTF‐A overexpression, while both MyoD and MyoG were up‐regulated in C2C12 cells lacking MRTF‐A (Figure 5C and D). We also placed MRTF‐A overexpressed C2C12 cells in differentiation medium for 0 and 3 days, respectively, and the extent of differentiation was quantified by immunofluorescence assay for MyoG and MyHC. As shown in Figure 5E and F, the differentiation was inhibited to a much lower extent in MRTF‐A overexpressed cell, as evidenced by fewer MyoG^+^ cells (Figure 5G) and reduced muscle fusion (myotube numbers and number of nuclei in MyHC^+^ myotubes) compared with control cells (Figure 5H), indicating that MRTF‐A is involved in the suppression of myogenic differentiation.

### 3.6 Lentivirus with MRTF‐A injection in mice attenuates satellite cell differentiation and muscle regeneration

**FIGURE 6:**
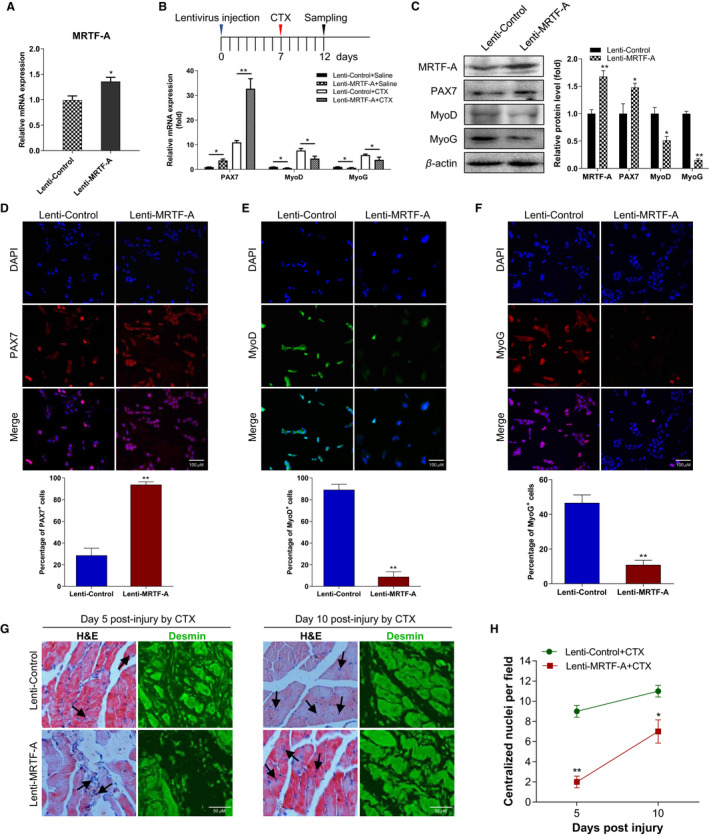
MRTF‐A overexpression by lentivirus injection decreases differentiating satellite cells and muscle regeneration. (A) The mRNA levels of the MRTF‐A gene were detected after lentivirus of Lenti‐Control and Lenti‐MRTF‐A injection for 3 days. (B) The mRNA expression of genes in regenerating muscle that was collected after CTX injury for 5 days (CTX‐D5). (C) Protein level of the regeneration involved genes in Lenti‐Control and Lenti‐MRTF‐A groups at CTX‐D5. (D–F) Satellite cells from the Lenti‐Control and Lenti‐MRTF‐A mice were isolated and cultured for 48 h in growth medium and immunostained for PAX7 (red), MyoD (green) and MyoG (red). Numbers of the PAX7^+^, MyoD^+^ and MyoG^+^ cells were shown below, respectively. Values are means ±SEM of triplicate experiments. Bar, 100 μm. (G) TA muscle from Lenti‐Control and Lenti‐MRTF‐A mice was injected with CTX and assayed for regeneration by H&E and by Desmin immunohistochemistry at days 5 and 10 post‐injury. Degenerating fibres are indicated by black arrowheads. Bar, 100 μm. (H) The number of centralized nuclei in Lenti‐MRTF‐A muscle compared with control muscle were quantitated at days 5 and 10 post‐injury. Analysis was performed on 5 mice and at least 5 sections from each animal. *p < 0.05, **p < 0.01

To further characterize the role of MRTF‐A in muscle development, we induced acute muscle damage by CTX injection in mice and assessed muscle regeneration capacity when MRTF‐A was overexpressed by lentivirus injection. The expression of MRTF‐A was confirmed at 3 days post‐lentivirus injection (Figure 6A). As expected, the PAX7 expression was increased, while MyoD and MyoG were markedly reduced when MRTF‐A was overexpressed in both CTX‐ and saline‐injected muscles (Figure 6B). Moreover, consistent results of the protein levels were also detected at 5 days after CTX injection (Figure 6C). To determine the effects of MRTF‐A on SCs quiescence and differentiation, we isolated the fresh SCs from MRTF‐A overexpressed or control muscles and performed the immunostaining for PAX7, MyoD and MyoG. The results showed that PAX7^+^ cells were higher (Figure 6D); however, the proportions of MyoD^+^ and MyoG^+^ cells were much lower in MRTF‐A overexpressed muscles compared to control muscles (Figure 6E and F), indicating that the SCs were primed less for differentiation. Moreover, the muscle regeneration was also evaluated by H&E or Desmin staining. As shown in Figure 6G, the control muscles were composed of more regenerating myofibres (centralized nuclei) than Lenti‐MRTF‐A‐injected muscle at 5 and 10 days post‐injury (Figure 6H). Similarly, Desmin‐positive myofibres were remarkably reduced in muscles with MRTF‐A overexpression at 5 days after injury and were still fewer in number and immature at 10 days after injury. Taken together, the *in vivo* data convincingly suggest that MRTF‐A activation impairs muscle regeneration by enhancing PAX7 and disallowing SCs commitment to differentiation.

### 3.7 MRTF‐A promotes the PAX7 expression by binding to its promoter region

**FIGURE 7:**
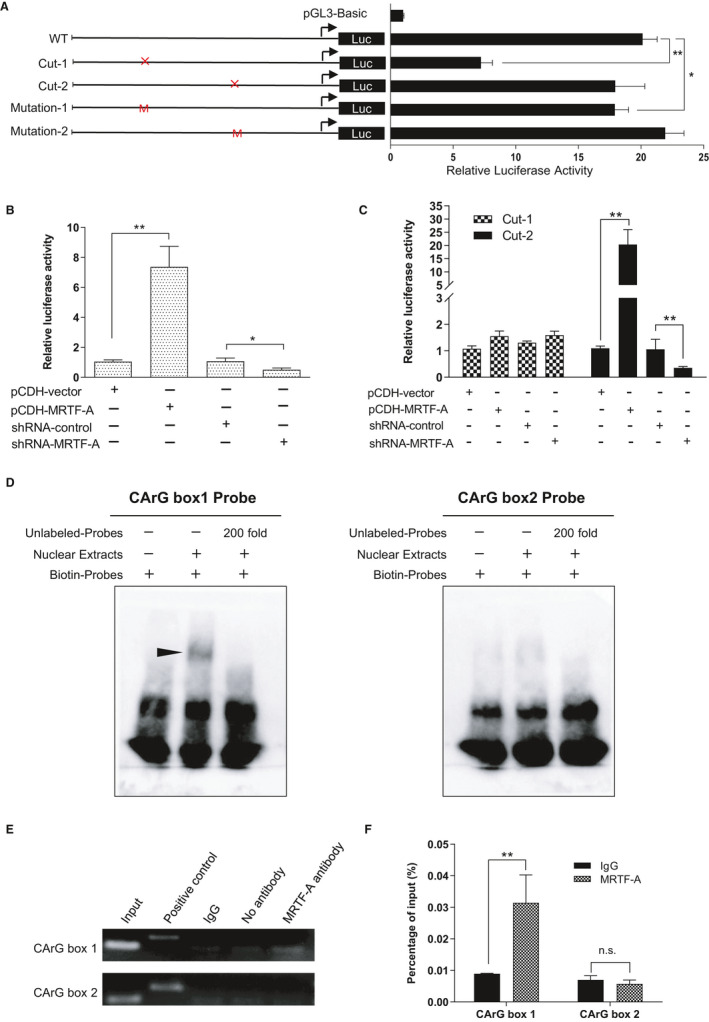
MRTF‐A regulates PAX7 expression by directly binding to the CArG box region of the *PAX7* promoter. (A) Promoter activity of the *PAX7* gene with WT, Cut‐1 (CArG box 1 cut‐down), Cut‐2 (CArG box 2 cut‐down), Mutation‐1 (CArG box 1 mutation) and Mutation‐2 (CArG box 2 mutation) promoters by dual‐luciferase reporter assay. (B) The effects of MRTF‐A on promoter activity of the *PAX7* gene. The pCDH‐vector, pCDH‐MRTF‐A, shRNA‐control and shRNA‐MRTF‐A were co‐transfected with dual‐luciferase reporter plasmids, respectively. (C) The effects of MRTF‐A on promoter activity of the *PAX7* gene with Cut‐1 or Cut‐2 promoter. The pCDH‐vector, pCDH‐MRTF‐A, shRNA‐control and shRNA‐MRTF‐A were co‐transfected with Cut‐1 or Cut‐2 plasmid, respectively. (D) Detection of interaction of MRTF‐A and CArG box 1 or CArG box 2 within the *PAX7* promoter by EMSA. A complete set of three reactions was performed using the nuclear extracts prepared from normal C2C12 cells. The 200‐fold of unlabeled probes were used as specific competitors to demonstrate that the signal shift observed results from specific protein: DNA interaction. Arrowhead shows the specific complex. (E) The binding of the MRTF‐A on CArG box of the *PAX7* gene promoter by ChIP assay. Histone H3 Antibody treatment was used as the positive control. (F) The percentage of input in IgG and MRTF‐A antibody–treated groups. *p < 0.05, **p < 0.01

Given that PAX7 contributes to muscle cell proliferation and differentiation by targeting the myogenic genes, such as *MyoD* and *MyoG*,^32^, ^33^ we hypothesized that MRTF‐A may governs cell commitment by directly regulating PAX7 expression. To test this possibility, we analysed that the promoter sequences of the *PAX7* gene and found two CArG box regions in ‐2345/‐2335 (Car1) and ‐2285/‐2276 (Car2) position of the *PAX7* promoter, respectively. Next, the reporter plasmids were constructed, containing either a wild‐type (WT), Car1 deleted (Cut‐1), Car2 deleted (Cut‐2), Car1 mutated (Mutation‐1) or Car2 mutated (Mutation‐2), and then sequenced the plasmids (Figure S4). Luciferase activity assay revealed that cells transfected with Cut‐1 or Mutation‐1 displayed significantly decreased transcriptional activity than WT (Figure 7A). MRTF‐A overexpression or knockdown markedly promoted or inhibited the luciferase activity of WT group, respectively (Figure 7B). However, the corresponding effects disappeared when MRTF‐A was co‐transfected with the Cut‐1 plasmid (Figure 7C). In addition, EMSA was performed to confirm the direct binding of MRTF‐A to the CArG box. To ensure the specific binding of MRTF‐A to the probe, unlabelled probes were added as competitors prior to the addition of the labelled probe. As shown in Figure 7D, a specific protein‐DNA complex was supershifted in cells using CArG box 1 probe. To characterize the interaction between nuclear extracts and the target sequence *in vivo*, we established a ChIP assay and revealed a parallel result to EMSA that an expected fragment was detected in the CArG box 1 group by PCR (Figure 7E) and qPCR (Figure 7F), while no fragment was generated by amplification targeting CArG box 2. All these data indicated that MRTF‐A could promote the *PAX7* transcription by directly binding to the CArG box 1 recognition element.

## 4 DISCUSSION

Cell‐based muscle regeneration is considered a heterologous or autologous approach to reconstitute and ameliorate muscle dystrophy.^34^, ^35^ However, the depletion of satellite cells can severely compromise muscle regeneration due to continuous rounds of muscle degeneration and repair. The process of self‐renewal of satellite cells is an important internal driver for maintaining and replenishing the muscle stem cell niche.^36^ Given the high expression patterns in quiescent satellite cells and their proliferating myoblast progeny, PAX7 functions as a molecular switch to instruct the myogenic fate of myoblast.^37^ Although a previous study reported that reduction or removal of PAX7 remarkably inhibits myoblast self‐renewal and is a key prerequisite for muscle cell differentiation,^38^ the molecular mechanism that regulates and retains the PAX7 level during muscle regeneration needs to be fully defined. In this study, we described a novel mechanism whereby MRTF‐A contributes to the elevated capacity of myoblast commitment towards differentiation by directly binding to the *PAX7* promoter.

Since MRTFs were discovered as the partner proteins of SRF to regulate skeletal muscle growth, maturation and regeneration,^39^, ^40^ substantial studies were established to explore the exact role and mechanisms of MRTF members. MASTR was reported to enhance the differentiation and impair proliferation by activating the transcriptional expression of *MyoD*.^26^ Selvaraj et al. revealed that MKL2/MRTF‐B contributed to the differentiation process from myoblasts to myotubes *in vitro* by regulating the skeletal α‐actin and α‐myosin heavy chain, while no activated event of MKL1/MRTF‐A (nucleus entry) was determined,^23^ suggesting that MRTF‐A may play contrasting roles with other MRTFs members. Herein, we report the declining expression of MRTF‐A at the late stages of muscle regeneration and investigated the inhibitory effects of MRTF‐A on myoblast differentiation. Our results were consistent with those of Holstein et al.^41^ who found that miR‐24‐3p/miR‐486‐5p‐mediated MRTF‐A reduction was observed during differentiation of murine C2C12 and primary human myoblast. The distinct functions may attribute to the alternative protein structures where MRTF‐A lack of the N‐terminal region, which is present in MRTF‐B, demonstrates an intensive interaction with SRF in mammalian cells.^42^ In addition, we also identified that MRTF‐A promoted the PAX7^+^/MyoD^−^ myoblast population proportion *in vitro* and significantly increased PAX7 positive cells in muscle injected by lentivirus *in vivo*. Extensive studies indicated that PAX7 is indispensable for SCs self‐renewal that account for supplementing the reservoir of SCs and driving muscle regeneration.^43^ Yu et al. reported that MRTF‐A had positive effects on self‐renewal capability in glioblastoma cells.^44^ However, whether MRTF‐A regulates SCs self‐renewal and the regulatory mechanisms have to be elaborated further.

Our study adds the MRTF transcriptional factor family to multiple regulators that drive the PAX7 expression in myoblast to affect adult muscle regeneration. Post‐transcriptional (ie miRNAs) or post‐translational (ie NEDD4) modifications have been described in previous studies to regulate the lineage specification and cell fate of the myoblast subpopulation by fine‐tuning PAX7 levels.^45^, ^46^, ^47^ MRTFs have been reported to couple extracellular signalling to adjust MyoD expression and alterations from SCs niche to SC activation.^48^ Our findings demonstrated that MRTF‐A and PAX7 presented reduced expressions in differentiated myoblasts and that MRTF‐A performed an operative role in regulating PAX7 levels. Precise manipulation of PAX7 expression is required for mediating SC heterogeneity, and the myoblast with PAX7 down‐regulation is primed more for myogenic differentiation through MyoD alteration.^33^ In addition, the PAX7‐MyoD protein ratios can determine the self‐renewal, proliferation and differentiation of myoblasts.^31^, ^49^ Here, we showed that MRTF‐A overexpression markedly reduced the PAX7^−^MyoD^+^ populations that towards differentiation, which raises the novel convincing evidence that the MRTF‐A acts as an effective sensor to regulate SCs cell fate by governing the MRTF‐A (high)‐PAX7 or MRTF‐A (low)‐PAX7‐MyoD signalling axis. Interestingly, many studies reported that the genes controlling SCs commitment and differentiation strongly contribute to muscular pathogenesis. For instance, miRNA‐431‐PAX7 axis accelerates muscle regeneration and ameliorates muscular dystrophy by regulating SCs heterogeneous population.^45^ Moreover, Wnt7a delays the muscular dystrophy progression by stimulating SCs expansion in *mdx* mice.^50^ Therefore, our findings about the role of MRTF‐A in muscle regeneration raises the possibility of therapeutic strategies that inhibition of MRTF‐A in myoblast, whether by dominant‐negative expression or antagonist treatment, will benefit patients with muscular disorders.

The regulation of SRF through CArG elements are required for the transcription of a wide variety of myogenic genes involved in differentiation and muscle regeneration.^51^, ^52^ Considering the ubiquitous expression of SRF, exploring the key factors that contribute to muscle‐specific transcription is critical to elucidate its regulatory mechanisms. In this study, we unveiled the novel functions of MRTF‐A as a transcriptional coactivator of PAX7 in myoblast proliferation and differentiation by interaction with CArG box elements within *PAX7* promoter. Further compelling experimental evidence in this study includes site‐directed mutagenesis, EMSA and the ChIP assay, which revealed that it is the distal CArG box (−2345 to −2335 bp), but not the proximal CArG box (−2345 to −2335 bp), interacting with MRTF‐A in the requirement for myoblast proliferation and differentiation. Interestingly, Kim et al.^53^ reported that the proximal CArG box within the α‐actin promoter was responsible for the Epc1 binding and synergistic activation of downstream genes that modulate skeletal muscle differentiation, suggesting that the coactivator of SRF presents a selective mode that accounts for the position of CArG elements. Our findings are relevant not only to establish the MRTF‐A as an important determinant in muscle regeneration, but also to clarify the regulatory mechanism of MRTF‐A through interacting with the *PAX7* promoter CArG box.

In conclusion, our study is, to our knowledge, the first to highlight the regulatory function of MRTF‐A in regulating myoblast commitment during muscle regeneration by targeting PAX7. Consistent expression profiling of MRTF‐A and PAX7 was demonstrated from acute muscle injury to coordinate repair. Overexpression of MRTF‐A enhances the proliferation process and impairs the myoblast differentiation and myotube formation. We further delineated the mechanism that MRTF‐A participates in the committed myoblast phenotypic regulation by directly binding to the CArG box element (Car1) of the *PAX7* promoter. Overall, the identification and assessment of the MRTF‐A function in this study represent an important advance in our understanding of the role of Myocardin family in satellite cell biology and open novel avenues to explore promising stem cell–based therapy for muscle degenerative diseases. The extent of the contribution of MRTF‐A in the molecular mechanism and regulatory networks of myogenic cell fate determination and muscle regeneration requires further exploration.

## CONFLICT OF INTEREST

The authors have declared no conflict of interest.

## AUTHOR CONTRIBUTIONS

**Ruhui Song:** Data curation (equal); Project administration (lead); Writing‐review & editing (supporting). **Shengnan Zhao:** Data curation (equal); Project administration (equal); Writing‐original draft (equal). **Yue Xu:** Data curation (equal); Project administration (equal); Writing‐original draft (equal). **Jian Hu:** Formal analysis (equal); Methodology (equal). **Shuangao Ke:** Data curation (equal); Methodology (equal); Visualization (equal). **Fan Li:** Investigation (equal); Methodology (equal). **Gaohui Tian:** Methodology (equal); Resources (equal). **Xiao Zheng:** Data curation (equal); Methodology (equal). **Jiajun Li:** Data curation (equal); Methodology (equal). **Lixing Gu:** Conceptualization (lead); Writing‐review & editing (lead). **Yao Xu:** Conceptualization (lead); Writing‐review & editing (lead).

## Supporting information

Figure S1

Figure S2

Figure S3

Figure S4

Table S1

## Data Availability

The data that support the findings of this study are available from the corresponding author upon reasonable request.
