# Supplementary material for: MRTF‐A regulates myoblast commitment to differentiation by targeting PAX7 during muscle regeneration
Source: J Cell Mol Med. 2021 Aug 4;25(18):8645–61. doi: 10.1111/jcmm.16820 (PMC8435411; doi:10.1111/jcmm.16820)
Supplement: Supplementary file 1 — Figure S1 [file JCMM-25-8645-s001.pdf]

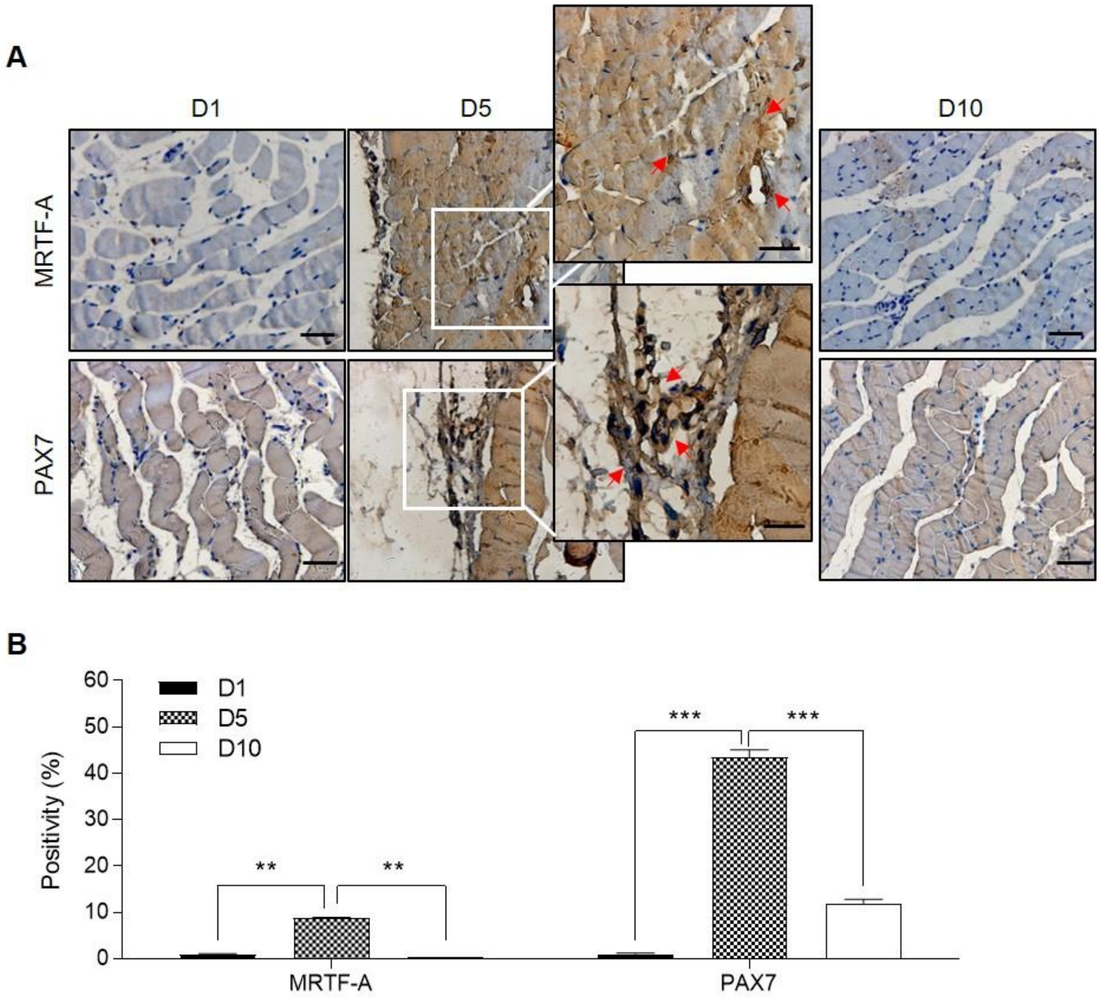

Figure S1. The expressions of MRTF-A and PAX7 are correlated with muscle regeneration after CTX injection. MRTF-A and PAX7 were detected in TA muscle of C57BL/6 mice by immunohistochemistry staining at D1, D5, and D10 after CTX injury. The red arrows show the positive regions for algorithm analysis. The scale bar represents 50 μm. The positivity of TA muscle for MRTF-A and PAX7 expression were quantified using Aperio Image Scope software. **p<0.01, ***p<0.001
