# Supplementary material for: MRTF‐A regulates myoblast commitment to differentiation by targeting PAX7 during muscle regeneration
Source: J Cell Mol Med. 2021 Aug 4;25(18):8645–61. doi: 10.1111/jcmm.16820 (PMC8435411; doi:10.1111/jcmm.16820)
Supplement: Supplementary file 2 — Figure S2 [file JCMM-25-8645-s004.pdf]

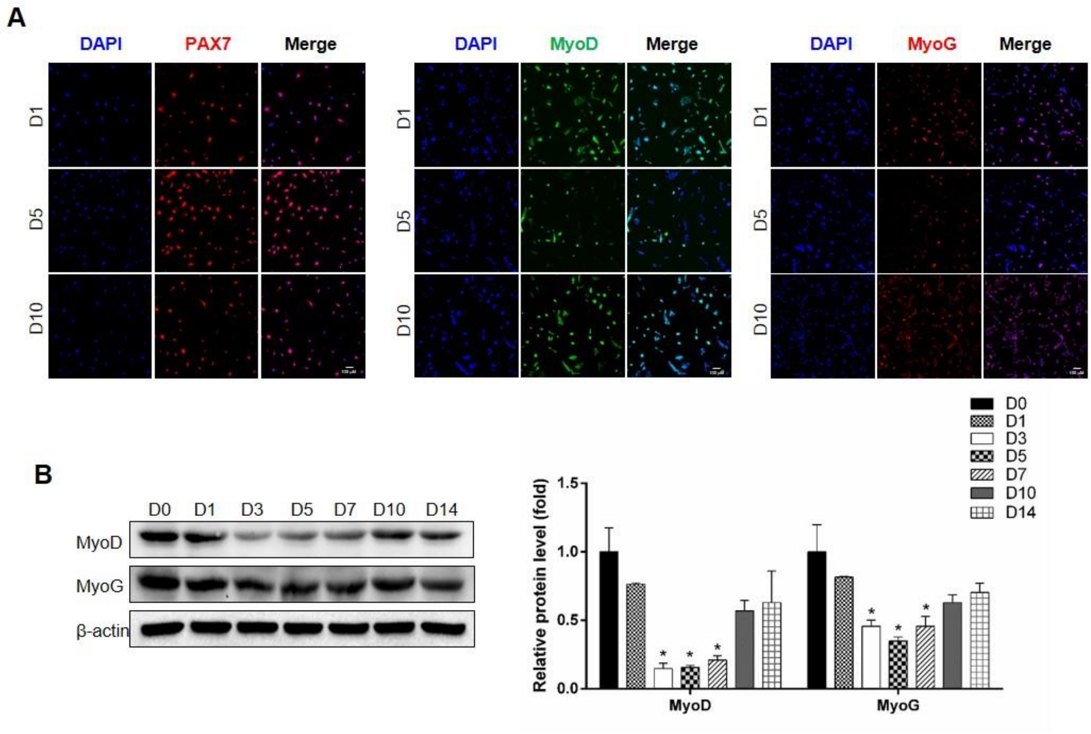

Figure S2. Detection of the muscle regeneration involving genes in C57BL/6 mice after CTX injection. (A) The determination of $PAX7^+$, $MyoD^+$, and $MyoG^+$ satellite cells isolated from TA muscles at D1, D5, and D10 after CTX injury by immunofluorescence staining. Scale bar: 200 μm. (B) The protein levels of MyoD and MyoG were detected by Western blot in TA muscles at D0, D1, D3, D5, D7, D10, and D14 after CTX injury. The β-actin was used to serve as a loading control. The densitometric quantification results were presented from three independent western blot experiments. $*p<0.05$
