# Supplementary material for: MRTF‐A regulates myoblast commitment to differentiation by targeting PAX7 during muscle regeneration
Source: J Cell Mol Med. 2021 Aug 4;25(18):8645–61. doi: 10.1111/jcmm.16820 (PMC8435411; doi:10.1111/jcmm.16820)
Supplement: Supplementary file 3 — Figure S3 [file JCMM-25-8645-s002.pdf]

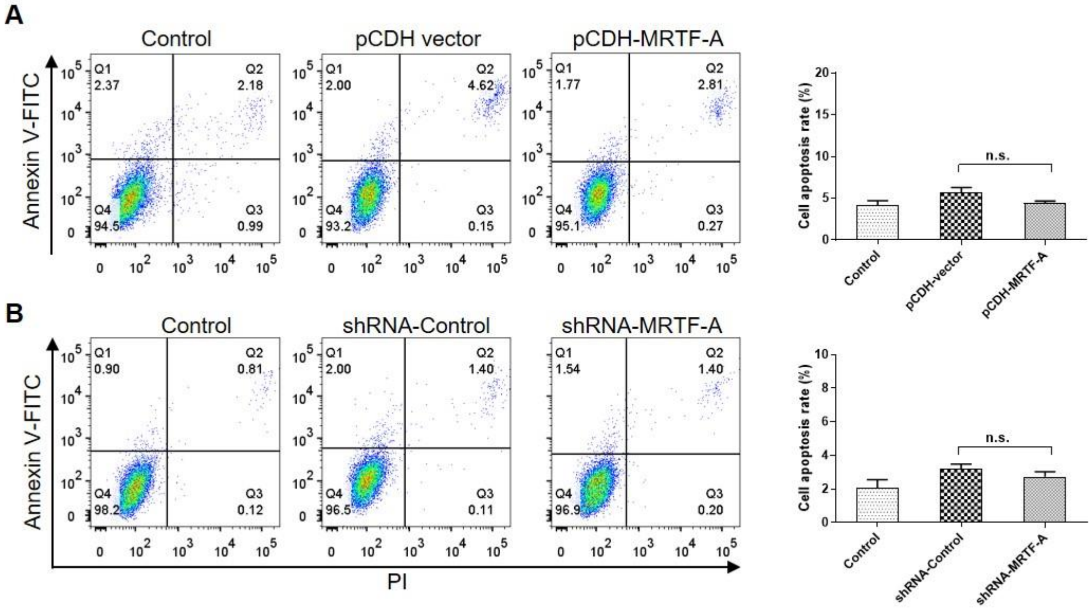

Figure S3. The effects of MRTF-A on cell apoptosis of myoblast. (A) The pCDH-vector and pCDH-MRTF-A were transfected into C2C12 cells, and the cell apoptosis was determined by Annexin V-FITC/PI binding followed by flow cytometry. (B) The MRTF-A was knocked down in C2C12 cells, and the cell apoptosis was determined by flow cytometry. Apoptosis rate (%) was analyzed from three independent experiments. Data are presented as means ± S.E.M., and n.s. shows no significance.
