# Supplementary material for: MRTF‐A regulates myoblast commitment to differentiation by targeting PAX7 during muscle regeneration
Source: J Cell Mol Med. 2021 Aug 4;25(18):8645–61. doi: 10.1111/jcmm.16820 (PMC8435411; doi:10.1111/jcmm.16820)
Supplement: Supplementary file 4 — Figure S4 [file JCMM-25-8645-s003.pdf]

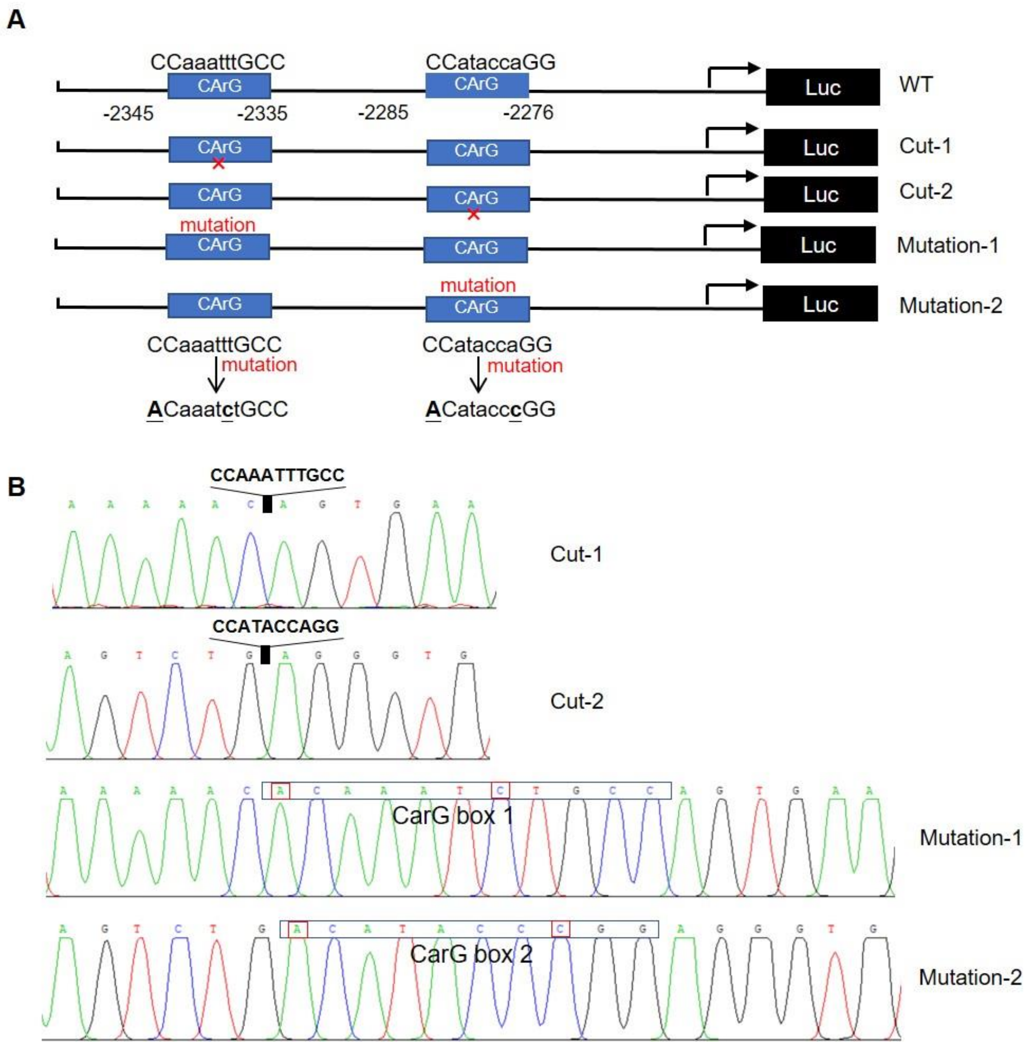


**Figure S4.** The design and construction of reporter plasmid with different mutations of CArG box in *PAX7* promoter. (A) Schematic diagram of CArG box 1/2 cutdown and mutation sites in *PAX7* gene. The red cross shows the deleted CArG box, and the underlined letters are pointed mutated bases by PCR. (B) The sequencing results of the constructed recombinant plasmids (Cut-1, Cut-2, Mutation-1, Mutation-2).
