# Supplementary material for: MRTF‐A regulates myoblast commitment to differentiation by targeting PAX7 during muscle regeneration
Source: J Cell Mol Med. 2021 Aug 4;25(18):8645–61. doi: 10.1111/jcmm.16820 (PMC8435411; doi:10.1111/jcmm.16820)
Supplement: Supplementary file 5 — Table S1 [file JCMM-25-8645-s005.docx]

**Table S1** Primers used in this study.

| Primer name | Genes | Primer sequences |
| --- | --- | --- |
| Primers for qPCR | *GAPDH* | F: AACTTTGGCATTGTGGAAGG |
|  |  | R: ACACATTGGGGGTAGGAACA |
|  | *PAX7* | F: CATGAACCCTGTCAGCAATG |
|  |  | R: CACTGTAGCCAGTGGTGCTG |
|  | *MRTF-A* | F: AACTGAAGTTGCGGTCCCTT |
|  |  | R: GAGGCCTCACCAGCCTTG |
|  | MyoD | F: CGCTCCAACTGCTCTGATGGCA |
|  |  | R: TGCTGCTGCAGTCGATCTCTCA |
|  | *MyoG* | F: CGGCTGCCTAAAGTGGAGAT |
|  |  | R: GCTGTGGGAGTTGCATTCAC |
|  | *Lix1* | F: GCAGCAGAAAGCCACCTT |
|  |  | R: GGGTCATCCGCATCATCT |
|  | *Mest* | F: GCAACCTGGTCATCGACA |
|  |  | R: TGATGGCCAGGACCTCTT |
|  | *PlagL1* | F: CTCCTACGCGTGTGACGATT |
|  |  | R: CGTGAGACTTGAGGTGGTCC |
|  | *Cipar1* | F: TTGGTCAGACCCAGGAAACT |
|  |  | R: CAATGGGACTGTTGGTGAAC |
|  | *Igfbp2* | F: CCCCCTGGAACATCTCTACTC |
|  |  | R: GGTATTGGGGTTCACACACC |
|  | *PCNA* | F: CCGAGACCTTAGCCACATTG |
|  |  | R: TCTCTATGGTTACCGCCTCC |
|  | *CyclinD* | F: TGTGCCACAGATGTGAAGTT |
|  |  | R: CAGTCCGGGTCACACTTG |
| Primers for knockdown or overexpression of MRTF-A | shRNA-*MRTF-A* | F:CCGGCATGGAGCTGGTGGAGAAGAACTCGAGTTCTTCTCCACCAGCTCCATGTTTTTGGTACCG |
|  |  | R:AATTCGGTACCAAAAACATGGAGCTGGTGGAGAAGAACTCGAGTTCTTCTCCACCAGCTCCATG |
|  | pCDH-*MRTF-A*^a^ | F: GC**TCTAGA**ATCATGCCGCCTTTGAAA |
|  |  | R: CG**GAATTC**CTACAAGCAGGAATCCCA |
| Primers for luciferase assay | *PAX7*-promoter-  WT | F: CAGGTACCAGTTACAGGGTTGGTATG |
|  |  | R: CTCAAGCTTGTTAAATATATGCTTG |
|  | *PAX7*-promoter-  Mutation-1^b^ | F: C**A**CAAAT**C**TGCCAGTGAAGAGCTA |
|  |  | R: CA**G**ATTTG**T**GTTTTTATGTGCAGGTG |
|  | *PAX7*-promoter-  Mutation-2^c^ | F: G**A**CATACC**C**GGAGGGTGTTGGTGGG |
|  |  | R: C**G**GGTATG**T**CAGACTTTTAGCGACTC |
|  | *PAX7*-promoter-  Cut-1 | F: TAAAAACAGTGAAGAGCTACCAA |
|  |  | R: TTCACTGTTTTTATGTGCAGGT |
|  | *PAX7*-promoter-  Cut-2 | F: AGTCTGAGGGTGTTGGTGGGGGTA |
|  |  | R: CACCCTCAGACTTTTAGCGACTC |
| Primers for EMSA | Biotin-Car1-probe | Sense:AAACCCAAATTTGCCAGTGAAGAGCTACCAAAC |
|  |  | Antisense:GTTTGGTAGCTCTTCACTGGCAAATTTGGGTTT |
|  | Car1-probe | Sense:AAACCCAAATTTGCCAGTGAAGAGCTACCAAAC |
|  |  | Antisense:GTTTGGTAGCTCTTCACTGGCAAATTTGGGTTT |
|  | Biotin-Car2-probe | Sense:CTGCCATACCAGGAGGGTGTTGGTGGGGGTAG |
|  |  | Antisense:CTACCCCCACCAACACCCTCCTGGTATGGCAG |
|  | Car2-probe | Sense:CTGCCATACCAGGAGGGTGTTGGTGGGGGTAG |
|  |  | Antisense:CTACCCCCACCAACACCCTCCTGGTATGGCAG |
| Primers for ChIP-qPCR | Car1 | F: ATTGACCCAAGACAGGCCTACA |
|  |  | R: TTAGCGACTCTCCTTTGTGTGG |
|  | Car2 | F: CCACACAAAGGAGAGTCGCTAA |
|  |  | R: GTTCTCCTTCAGTCTTCCATCTGT |

a, the underlined base showed the restriction sites of *Kpn* I and *Hin*d III, respectively.

b and c, the underlined base showed mismatches to disrupt CArG box sites.
